# Executive Function Changes before Memory in Preclinical Alzheimer’s Pathology: A Prospective, Cross-Sectional, Case Control Study

**DOI:** 10.1371/journal.pone.0079378

**Published:** 2013-11-18

**Authors:** Michael G. Harrington, Jiarong Chiang, Janice M. Pogoda, Megan Gomez, Kris Thomas, Sarah DeBoard Marion, Karen J. Miller, Prabha Siddarth, Xinyao Yi, Feimeng Zhou, Sherri Lee, Xianghong Arakaki, Robert P. Cowan, Thao Tran, Cherise Charleswell, Brian D. Ross, Alfred N. Fonteh

**Affiliations:** 1 Molecular Neurology Program, Huntington Medical Research Institutes, Pasadena, California, United States of America; 2 Keck School of Medicine at the University of Southern California, Los Angeles, California, United States of America; 3 Fuller Graduate School of Psychology, Pasadena, California, United States of America; 4 David Geffen School of Medicine, UCLA, Los Angeles, California, United States of America; 5 Department of Chemistry and Biochemistry, California State University, Los Angeles, California, United States of America; 6 Dept of Neurology, Stanford University School of Medicine, Stanford, California, United States of America; 7 Magnetic Resonance Program, Huntington Medical Research Institutes, Pasadena, California, United States of America; Nathan Kline Institute and New York University School of Medicine, United States of America

## Abstract

**Background:**

Early treatment of Alzheimer’s disease may reduce its devastating effects. By focusing research on asymptomatic individuals with Alzheimer’s disease pathology (the preclinical stage), earlier indicators of disease may be discovered. Decreasing cerebrospinal fluid beta-amyloid_42_ is the first indicator of preclinical disorder, but it is not known which pathology causes the first clinical effects. Our hypothesis is that neuropsychological changes within the normal range will help to predict preclinical disease and locate early pathology.

**Methods and Findings:**

We recruited adults with probable Alzheimer’s disease or asymptomatic cognitively healthy adults, classified after medical and neuropsychological examination. By logistic regression, we derived a cutoff for the cerebrospinal fluid beta amyloid_42_/tau ratios that correctly classified 85% of those with Alzheimer’s disease. We separated the asymptomatic group into those with (n = 34; preclinical Alzheimer’s disease) and without (n = 36; controls) abnormal beta amyloid_42_/tau ratios; these subgroups had similar distributions of age, gender, education, medications, apolipoprotein-ε genotype, vascular risk factors, and magnetic resonance imaging features of small vessel disease. Multivariable analysis of neuropsychological data revealed that only Stroop Interference (response inhibition) independently predicted preclinical pathology (OR = 0.13, 95% CI = 0.04–0.42). Lack of longitudinal and post-mortem data, older age, and small population size are limitations of this study.

**Conclusions:**

Our data suggest that clinical effects from early amyloid pathophysiology precede those from hippocampal intraneuronal neurofibrillary pathology. Altered cerebrospinal fluid beta amyloid_42_ with decreased executive performance before memory impairment matches the deposits of extracellular amyloid that appear in the basal isocortex first, and only later involve the hippocampus. We propose that Stroop Interference may be an additional important screen for early pathology and useful to monitor treatment of preclinical Alzheimer’s disease; measures of executive and memory functions in a longitudinal design will be necessary to more fully evaluate this approach.

## Introduction

Cognitively healthy (CH) people with preclinical Alzheimer’s disease (AD) have AD pathology many years before their first symptoms, and this preclinical phase is currently recognized with a combination of brain imaging, genetic, and cerebrospinal fluid (CSF) biomarkers [1]. Age-appropriate CSF beta amyloid_42_ (Aß_42_) and total tau (Tau) cutoffs [2] and the Aß_42_/Tau ratio [3] are diagnostic biomarkers of AD. Based on staging of preclinical AD with CSF biomarkers, Jack et al. recently recommended definitions of stage 1 and stage 2 as lowered CSF beta Aß_42_ and lowered Aß_42_ with rising Tau, respectively [4], [5]. When CSF Tau changes in the absence of a decrease in Aß_42_, these authors suggested the term SNAP (non-AD pathophysiology), recognizing that this CSF profile is not consistent with AD pathology [5]. These stages reflect the general consensus of how CSF Aß_42_ and Tau evolve in AD. Compared to the biomarkers themselves, the Aß_42_/Tau ratio has been found to be more sensitive in detecting symptomatic AD and differentiating it from fronto-temporal dementia [6], [7]. Therefore, it is possible that the earliest preclinical AD may also be detected more sensitively and specifically by this biomarker ratio rather than from the individual biomarkers.

The earliest symptoms from AD pathology are non-specific and may not be recognized [8], but the pathology is likely to progress from non-symptomatic to subjective cognitive impairment [9], then to mild cognitive impairment (MCI) [10], and finally to dementia [11]. Current research is more clearly defining early symptomatic AD [12]. A better understanding and diagnosis of the preclinical phase is important since interventions, including those that have been shown to be unsuccessful in later stages, might delay clinical deterioration if given earlier [13].

To investigate when AD biomarkers might change, Bateman et al. studied mutation carriers of autosomal dominant AD to take advantage of the more reliable predictability of future disease in this population and estimated that Aß_42_ decreased as early as 25 years before expected symptom onset [14]. These authors showed that the progressive neuropathology subsequently involved brain amyloid deposition, followed by increasing brain atrophy and increasing CSF Tau levels, after which episodic memory impairment and cerebral glucose hypometabolism were detected. Only then did symptoms appear and progress to global cognitive impairment.

Decreasing levels of CSF Aß_42_ and amyloid imaging also identify late-onset preclinical AD [15], but prediction of time to dementia onset is not as reliable as in the early onset autosomal dominant AD. Furthermore, compared to the extensive knowledge of the dementia stage [16]–[18], little is known about late onset preclinical AD beyond the aforementioned evolution of pathology biomarkers. In autopsy studies designed to define the pathology of AD, investigators found that the first intraneuronal neurofibrillary abnormalities were limited to the hippocampus, and next progressed to limbic, then to isocortical regions [19]. Extracellular amyloid deposits started in basal portions of the isocortex and became more generalized over time, but in contrast to the neurofibrillary changes, the hippocampal amyloid deposits appeared only at later stages [19]. This research also demonstrated that extracellular amyloid usually appeared before any intraneuronal neurofibrillary changes. The location of the pathological extracellular amyloid has been replicated in humans by amyloid imaging *in vivo* [20]–[22]. Direct imaging of neurofibrillary pathology has not yet been possible, though methods to study this are under development [23], [24].

Diagnostic criteria for preclinical AD are new but studies suggest both memory and executive functions are affected [25], [26]. Further neuropsychological assessment of individuals with preclinical AD may give clues to the brain functions and pathology locations that are first affected in AD. The goals of this cross-sectional study were to use a derived CSF Aß_42_/Tau cutoff level to separate CH study participants into two subgroups, those with and those without preclinical AD, and to evaluate differences in neuropsychological measures between these two subgroups. We recruited an older population so as to include a large proportion of preclinical AD participants.

## Materials and Methods

### Human Participants

The Institutional Review Board of the Huntington Hospital, Pasadena, CA, approved the protocol and consent forms for this study (HMH-99-09) and all study participants gave written, informed consent.

Participants over age 70 years were recruited through newspaper articles, visits to senior centers and assisted living facilities, and word of mouth. Inclusion criteria included age 70–100 years and presenting as CH, having mild cognitive impairment (MCI), or having AD (defined below). Exclusion criteria included taking strong anticoagulants and contraindications for MRI or lumbar puncture. Consistent with the Uniform Data Set [27], the following data were obtained from each individual within one month of informed consent: structured clinical interview; complete prescription and over-the-counter medication and nutritional supplements history; and physical examination with a focus on neurological and cardiovascular systems. Participants self-scored physical and intellectual activity (separately) over the past year on a 1 to 4 scale, where 1 = minimal, 2 = moderate, 3 = active, and 4 = very active. Body mass index (BMI) was calculated from measured height and weight. A physician or nurse, blinded to diagnostic classification, measured diastolic and systolic blood pressures (mm Hg) with study participants seated and rested on two occasions during the workup, and the pressures were averaged. Laboratory workup to exclude conditions that might affect cognition included blood CBC, ANA, ANCA, phosphorus, vitamin B12, folate, Lyme antibody detection by EIA, HIV, syphilis, comprehensive metabolic panel, and thyroid function panel; and urinalysis screened for protein, blood, leukocytes, nitrite, glucose, ketone, pH, specific gravity, bilirubin and urobilinogen. Fasting blood glucose and lipids (mg/dl) were determined by routine methods immediately before CSF collection (below). MRI was performed on a 1.5 T GE scanner to exclude significant vascular or neoplastic disorders. To assess small vessel disease (SVD) [28], two qualified, blinded readers performed an analysis based on that recently proposed by Wardlaw et al [28] in which 6 items contribute to the MRI diagnosis of SVD. In the absence of an assessment for “cerebral microbleeds” (no susceptibility weighted imaging was performed), we investigated 5 contributing SVD features: “recent small subcortical infarcts”, “lacunes of presumed vascular origin”, “white matter hyperintensities of presumed vascular origin” (uncorrected for brain volume), “perivascular spaces”, and “brain atrophy”. We assigned 0 if absent or 1 if present for each feature, for a maximum score of 5. Hippocampal volumes relative to whole brain volume were derived using FreeSurfer software version 5.0 (www.surfer.nmr.mgh.harvard.edu). Proton magnetic resonance spectroscopy (MRS) was performed on posterior cingulate grey (PCG) and left parietal white matter (PROBE-P TE = 35 milliseconds, TR = 1.5 seconds, 8 mL voxel, 128 averages). Results of PCG-MRS were expressed as NAA/mI following observer independent spectral analysis using SAGE (GE Healthcare) and compared with an age-appropriate data base of normative results in >100 subjects [noting that N-acetyl aspartate (NAA), a putative neuronal biomarker, is reduced by dementing diseases, including MCI and AD, and that myo-inositol (mI), a likely biomarker of glia, is correspondingly increased [29]–[31].

Specific testing included the Functional Activity Questionnaire [32] (FAQ); Mini-Mental State Exam [33]; Montreal Cognitive Assessment [34]; Geriatric Depression Scale [35]; Clinical Dementia Rating [36] (CDR); and neuropsychological battery. The battery included: Wechsler Test of Adult Reading [37] (WTAR); Wechsler Adult Intelligence Scales-III [38] (WAIS-III) to establish current full-scale intelligence quotient, verbal intelligence quotient and performance intelligence quotient [39]; WAIS-III Digit Span, Letter Number Sequencing, Matrix Reasoning, Digit Symbol, Similarities, Information, Block Design, Arithmetic, Picture Completion; Wechsler Memory Scale-III [40] Logical Memory I and II (WMS-III, LM I & LM II); California Verbal Learning Test-II & Delay [41] (CVLT-II); Rey Osterreith Complex Figure- Copy, Delay, & Recognition [42] (Rey-O Copy), Judgment of Line Orientation [43] (JLO); Controlled Oral Word Association Test [44], Animals [45]; Boston Naming Test [43]; Trail-making Test A & B [46]; Stroop Color-Word and Word Interference Tests [47]; Purdue Pegboard (bimanual); Tower from Delis-Kaplan Executive Functioning System [48] (D-KEFS); and Brief Visual Memory Test, Revised [49], [50] (BVMT-R) and BVMT-R Delay.

Composite neurocognitive domain z scores were the means of summed individual test z scores for each of the following domains: Attention/Concentration/Working Memory = WAIS-III Digit Span, WAIS-III LN sequencing, WAIS-III Arithmetic; Psychomotor/Processing Speed = WAIS-III Digit Symbol, TRAILS A, Stroop Color Naming, Stroop Word Reading; Language = COWAT, Animals, BNT, WAIS-III Information; Delayed memory = LMII, CVLT-LDFR Correct, BVMT-R Delay, Rey O 3 minute delay; Verbal Memory = LMI, LM II, CVLT Trials 1–5, CVLT LDFR; Nonverbal Memory = BVMT-R, BVMT-R Delay, Rey-O 3 Minute Delay; Encoding = LM1, BVMT-R; Visuospatial/Construction = WAIS-III Block Design, WAIS-III Picture Completion, JLO; Comprehensive Executive = WAIS-III Matrix Reasoning, WAS-III Similarities, COWAT-FAS, Animals, DKEFS Tower, TRAILS B, Stroop Interference; Core Executive = DKEFS-Tower, Trails B, Stroop Interference; Motor Functioning = Purdue Pegboard.

After the complete assessment, classification of CH participants was based primarily on being asymptomatic with a CDR and FAQ total score of zero, and neuropsychological measures that scored within at least one standard deviation of the mean for their age and education according to published normative values, and did not meet criteria for MCI [10] (MCI amnestic or nonamnestic) or dementia [11], [51], [52]. MCI and clinically probable AD were diagnosed in study participants that fulfilled the current criteria [10], [11], [51], [52]. Clinical diagnoses of “other” dementias, necessary to have the most accurate diagnosis of the AD dementia group, were based on the referenced consensus criteria as follows: Lewy Body dementia [53], behavior variant frontotemporal dementia [54], and vascular or mixed dementia [55]. Clinically probable dementia (AD, Lewy Body disease, frontotemporal dementia, vascular dementia, or mixed dementia), MCI, and CH classification were assigned from the aforementioned workup after triple scoring of neuropsychological test results by research staff and clinical conferencing by a minimum of 3 faculty clinicians.

### Apoε Genotype

Blood peripheral lymphocytes and standard methods for Apoε genotyping [56] were used.

### CSF Cell Count, Total Protein, and Aß_42_ and Tau

After an overnight fast, lumbar CSF was obtained between 8∶00 am and 10∶00 am and was immediately examined for cells and total protein. Cells were counted in a hemocytometer after trypan blue staining. Total protein concentrations were determined with the fluorescent Quant-iT™ protein assay kit (Invitrogen/Molecular Probes, Eugene, OR) with bovine serum albumin (0–500 ng/ml) as a standard for quantification. Fluorescence (excitation at 470 nm and emission at 570 nm) was measured using a Gemini XPS Dual-Scanning Microplate Spectrofluorometer and data analyzed using SoftMax® Pro software (Molecular Devices, Sunnyvale, CA). The remaining CSF was stored in 1 mL aliquots (polypropylene cryo vials, # V9380-100EA, Sigma-Aldrich) at −80°C until thawed for Aß_42_ and Tau assay using a sandwich enzyme-linked immunosorbent assay kit (Innotest β-amyloid_(1–42)_ and Innotest hTAU-Ag, Innogenetics, Gent, Belgium) according to the manufacturer’s protocol. In brief, to determine the concentration of Aß_42_, 25 µL of CSF sample and standards were added into the monoclonal antibody (21F12) precoated plate and incubated with biotinylated antibody (3D6). All assays were performed in the same week from CSF aliquots that had never been re-frozen, were collected within a 2 year period and stored in a freezer that was known from monitoring to have not warmed above −75°C. Forty CSF samples were analyzed per plate in duplicate, blind to clinical diagnosis, with 8 standards in duplicate. The Aß_42_ concentration was determined from the standard curve, in the range between 125 and 2000 pg/mL, and the lower limit of detection for this assay was 50 pg/mL. The average coefficient of variation for all samples was 6.7% and the median was 5.3%. In the Tau assay, 25 µL of CSF sample and standards were added in duplicate into the monoclonal antibody (AT120) precoated plate and incubated overnight with two biotinylated Tau-specific antibodies (HT7 and BT2). The concentration of Tau was determined from the standard curve, in the range between 75 and 1200 pg/mL. The lower limit of detection for this assay was 59.3 pg/mL.

### Statistical Methods

Multinomial logistic regression was used to determine a cutoff for the ratio Aß_42_/Tau that would provide 85% sensitivity in discriminating AD from CH and MCI participants; specifically, the cutoff value was the solution for x in the equation:
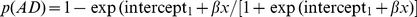
(1)where p(AD) was the probability of “AD” that yielded 85% sensitivity, intercept_1_ was the estimated intercept for the equation that predicted the outcome of “CH or MCI,” and β was the estimated coefficient for the predictor x = Aß_42_/Tau.

Differences between clinical groups were tested using two-sided t-tests (except when we tested for the known decrease from neurodegenerative disease in a one-sided t-test for NAA/mI ratios) or Wilcoxon rank sum tests for continuous and ordinal variables and Fisher’s exact or chi squared tests for categorical variables. Multivariable analysis was used to evaluate tests of neuropsychological performance as independent predictors of clinical group membership and included only participants with non-missing data for all independent variables.

Maximum likelihood estimates of odds ratios (ORs) and 95% confidence intervals (CIs) were calculated using logistic regression. The “best-fitting” model was derived by a manual variable selection technique that began with all variables that were potential predictors in the model and proceeded to eliminate variables in succession of highest to lowest p-values that were non-significant; i.e., p≥0.05. Any variable that had been removed was eligible to re-enter if removal of a subsequent variable resulted in the removed variable becoming significant. The variable selection procedure ended when all remaining variables in the model and none of the variables not in the model were significant. A receiver operating characteristic ROC curve was used to describe model fit.

To minimize the potential for type 1 error, we first analyzed clinical domains to arrive at a subset that significantly predicted group membership in a multivariable model. We then analyzed individual tests within these domains. To minimize collinearity in multivariable analyses, correlations were assessed for every pair of potential predictor variables, with the criterion for “high” correlation being r^2^≥0.50, where r^2^ was the coefficient of determination from the linear regression of one member of the pair on the other member of the pair. For pairs that were highly correlated, the member most related to outcome was retained for multivariable analysis.

Statistical analyses were done using SAS v 9.2 (SAS Institute, Inc., Cary, NC). All statistical tests were performed at the 0.05 significance level.

## Results

**Figure 1:**
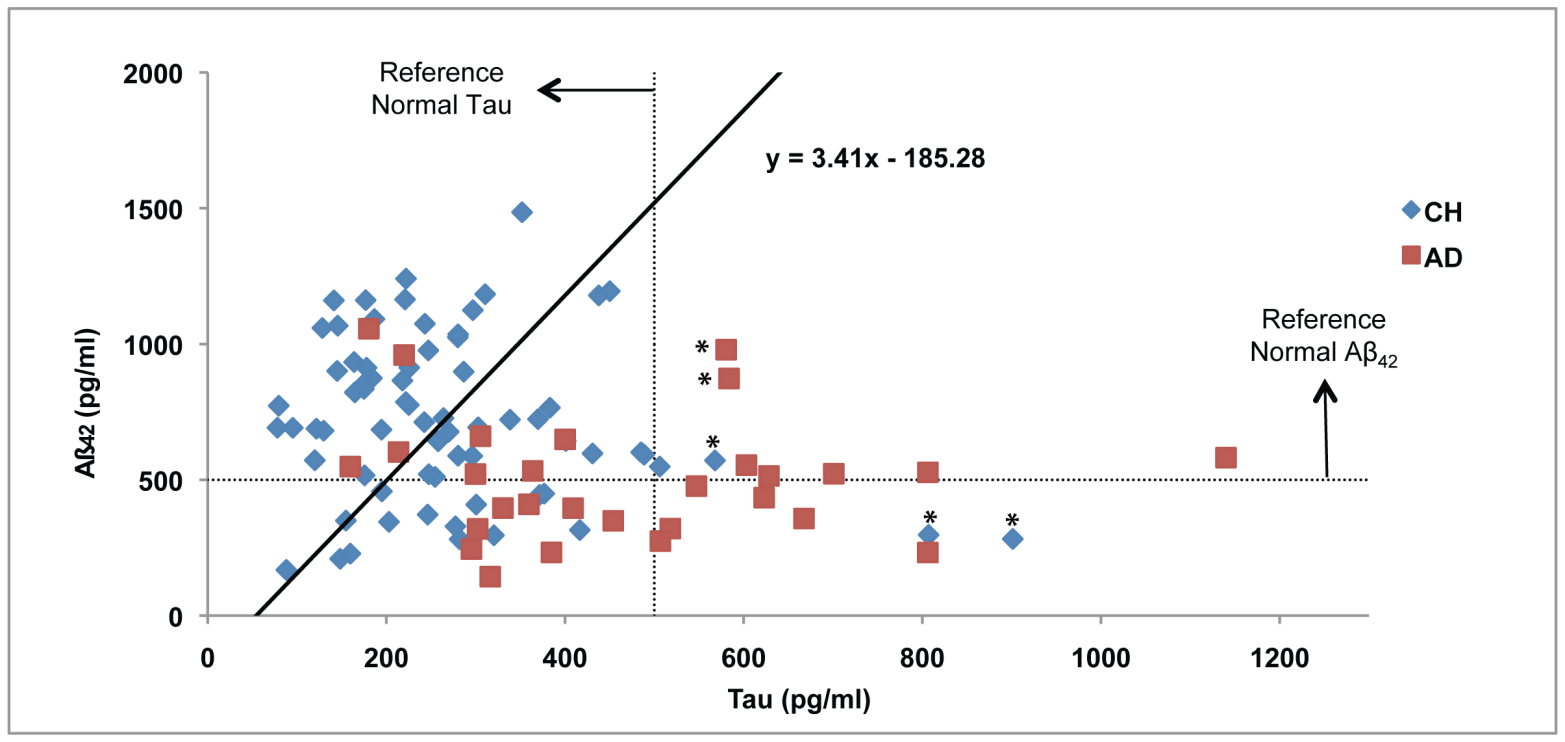
Prediction of AD from individual CSF Aß_42_ and Tau concentrations. Levels of Tau are plotted against Aß_42_ for each CH (blue diamond) or AD (red square) individual. The diagonal line indicates the regression-derived cutoff for the ratio Aß_42_/Tau that provides 85% sensitivity in discriminating AD participants from CH and MCI participants (MCI participants were used in the regression analysis but their results were excluded in the figure for simplicity): above the diagonal line is healthy, below is AD with 85% probability. The horizontal and vertical (dotted) lines mark the cutoff values for Aß_42_ (normal above) and Tau (normal to the left), respectively, from age-appropriate controls [2]. The * denotes individual values that are discussed in the Results text.

149 participants consented and were classified as having dementia (n = 39), having MCI (n = 40), or being CH (n = 70). Ten of the dementia participants were classified as Lewy Body dementia, behavior variant frontotemporal dementia, vascular dementia, or mixed dementia and were excluded from further analysis; the remaining 29 dementia participants had clinically probable AD. The Aß_42_/Tau ratio cutoff was calculated as 2.7132 for the total study population (sensitivity = 85%, specificity = 64%), comprised of 29 AD, mean (SD) age = 77 (10); 40 MCI, mean (SD) age = 77 (7); and 70 CH participants, mean (SD) age = 77 (7). We identified two subgroups from the 70 CH participants (Fig. 1): 36 had a ratio above or on the cutoff, i.e., those with Normal Aß_42_/Tau proteins (“CH-NAT”), and 34 had a ratio below the cutoff, i.e. those with Pathological Aß_42_/Tau proteins (“CH-PAT”). We thus defined that CH-PAT participants had preclinical AD.

To determine the preclinical stages of the CH-PAT participants, we examined the individual measurements compared with age-appropriate Aß_42_ and Tau cutoffs [2] (Fig. 1). Most CH-PAT participants were in stage 1 [4], with Tau levels below 500 pg/mL. The exceptions were three CH-PAT participants (marked with * in Fig. 1) with Tau above 500 pg/mL (a fourth had borderline elevated Tau). Two of the exceptions had abnormal Aß_42_, consistent with stage 2 preclinical AD [4]; these participants were 80 and 77 years of age, had higher-level educations (college degrees), and had Apoε 3/4 genotype. The third exception had normal Aß_42_ with a Tau of 568 pg/mL, consistent with SNAP [5], was 84 years of age, had higher-level education, and had Apoε 2/3 genotype.

Examining the AD group, half of the participants did not have elevated Tau, consistent with early pathology. Two clear outliers in the AD group had elevated Tau with normal Aß_42_ (marked with * in Fig. 1): they both had typical clinical AD but, since they died without post-mortem, another diagnosis was possible.

Clinical groups in Tables 1–3 summarize baseline characteristics and medication and supplement use. Education as well as activity levels differed significantly by clinical group: AD participants had lower levels of education, while self-reported physical and intellectual activity declined with increasing cognitive impairment. There was no evidence of differences by clinical group in any of the other variables in Tables 1–3.

**Table 1:** Demographics by clinical group.

Parameter		CH-NAT	CH-PAT	AD	p-value^1^
**Age (years)**	N	36	34	29	0.686
	Mean (SD)	76.4 (7.05)	78.0 (6.46)	77.4 (9.57)	
	Median	75.0	78.5	79.0	
	Min, Max	63, 89	63, 89	47, 91	
**Gender [n (%)]**	Female	22 (61.1)	21 (61.8)	16 (55.2)	0.872
	Male	14 (38.9)	13 (38.2)	13 (44.8)	
**Education Level Score** 2	N	36	34	28	**<0.001**
	Mean (SD)	6.4 (1.73)	6.1 (2.12)	4.1 (2.12)	
	Median	6.5	6.0	4.0	
	Min, Max	2, 8	1, 8	1, 8	
**BMI (kg/m** 2 **) [n (%)]**	<20 (underweight)	3 (8.3)	0 (0.0)	1 (3.8)	0.795
	20–25 (normal)	15 (41.7)	12 (36.4)	10 (38.5)	
	25.1–30 (overweight)	14 (38.9)	16 (48.5)	12 (46.2)	
	>30 (obese)	4 (11.1)	5 (15.2)	3 (11.5)	
	Not available	0	1	3	

1 Fisher’s exact test for categorical variables, t-test or Wilcoxon rank sum test for continuous variables.

2 Education scores: 1 = <high school, 2 = high school diploma, 3 = some college, 4 = 2-year college degree, 5 = 4-year college degree, 6 = some post-graduate college, 7 = post-graduate degree. CH-NAT = cognitively healthy with normal CSF amyloid/Tau ratio. CH-PAT = cognitively healthy with pathological CSF amyloid/Tau ratio.

**Table 2:** Activity scores and Apo E genotype by clinical group.

Parameter		CH-NAT	CH-PAT	AD	p-value^1^
**Intellectual Activity Score** 2	N	35	33		**0.046**
	Mean (SD)	3.0 (0.51)	2.6 (0.78)		
	Median	3.0	3.0		
	Min, Max	2, 4	1, 4		
**Physical Activity Score**	N	35	33	27	**<.0001**
	Mean (SD)	2.9 (0.63)	2.5 (0.62)	2.1 (0.62)	
	Median	3.0	3.0	2.0	
	Min, Max	2, 4	1, 3	1, 3	
**Apo E Genotype [n (%)]**	2/2	0 (0.0)	1 (3.1)	0 (0.0)	0.455
	2/3	2 (5.7)	5 (15.6)	1 (3.4)	
	3/3	12 (34.3)	9 (28.1)	6 (20.7)	
	2/4	1 (2.9)	1 (3.1)	0 (0.0)	
	3/4	19 (54.3)	15 (46.9)	19 (65.5)	
	4/4	1 (2.9)	1 (3.1)	3 (10.3)	
	Not available	1	2	0	

1 Fisher’s exact test for categorical variables, t-test or Wilcoxon rank sum test for continuous variables.

2 Not collected for AD subgroup. CH-NAT = cognitively healthy with normal CSF amyloid/Tau ratio. CH-PAT = cognitively healthy with pathological CSF amyloid/Tau ratio.

**Table 3:** Medication and supplement use by clinical group.

Parameter		CH-NAT	CH-PAT	AD	p-value^1^	
**# Prescription Medications**	N	36	34	29	0.773	
	Mean (SD)	3.5 (2.57)	3.2 (2.31)	3.5 (2.13)		
	Median	3.0	3.0	3.0		
	Min, Max	0, 10	0, 10	0, 8		
**On Lipid Modifiers [n (%)]**	No	25 (69.4)	23 (67.6)	19 (65.5)	0.963	
	Yes	11 (30.6)	11 (32.4)	10 (34.5)		
**On NSAIDs [n (%)]**	No	19 (52.8)	16 (47.1)	17 (58.6)	0.696	
	Yes	17 (47.2)	18 (52.9)	12 (41.4)		
**On HRT [n (%)]**	No	31 (86.1)	32 (94.1)	23 (79.3)	0.217	
	Yes	5 (13.9)	2 (5.9)	6 (20.7)		
**# Anti-Hypertensive Medications [n (%)]**	0	11 (30.6)	17 (50.0)	12 (41.4)	0.894	
	1	15 (41.7)	12 (35.3)	7 (24.1)		
	2	7 (19.4)	4 (11.8)	6 (20.7)		
	>2	3 (8.3)	1 (2.9)	4 (13.8)		
**# OTC Medications [n (%)]**	0	15 (41.7)	7 (20.6)	14 (48.3)	0.704	
	1	17 (47.2)	19 (55.9)	13 (44.8)		
	>1	4 (11.1)	8 (23.5)	2 (6.9)		
**# Supplements**	N	36	34	29	0.180	
	Mean (SD)	3.8 (3.23)	4.9 (3.02)	3.9 (3.86)		
	Median	4.0	4.0	3.0		
	Min, Max	0, 15	1, 16	0, 12		

1 Fisher’s exact test for categorical variables, t-test or Wilcoxon rank sum test for continuous variables. CH-NAT = cognitively healthy with normal CSF amyloid/Tau ratio. CH-PAT = cognitively healthy with pathological CSF amyloid/Tau ratio.

**Figure 2:**
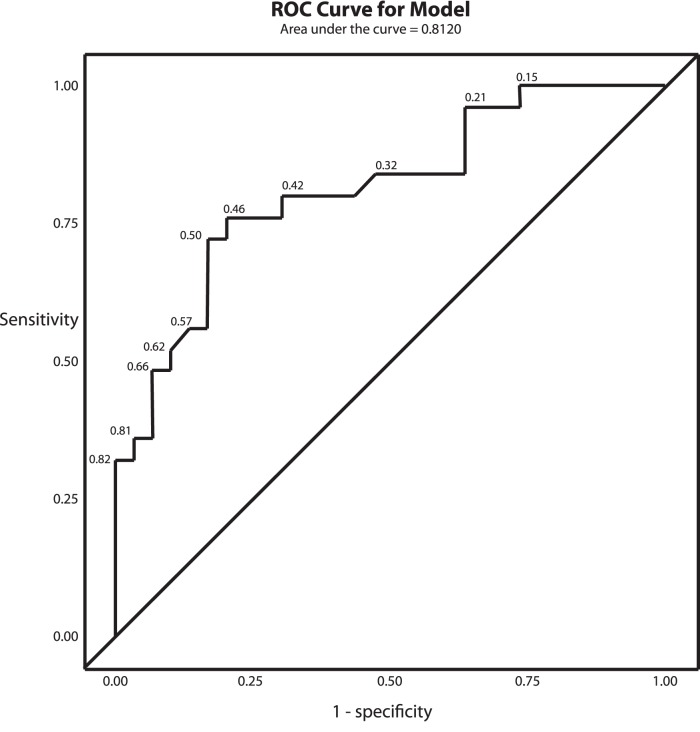
Receiver operating characteristic (ROC) curve associated with Stroop Interference as a discriminator between CH-NAT and CH-PAT. Area under the curve = 0.81. The diagonal line represents no predictive ability of the model. Steps of the step function represent different cut-off points (labeled) for the predicted probability that a given observation is from a CH-PAT subject.

**Figure 3:**
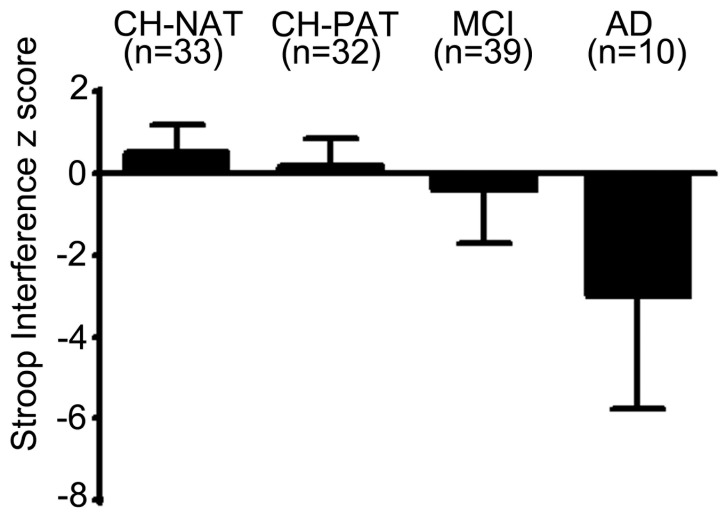
Stroop Interference z scores by clinical group. Mean (SD) Stroop Interference z scores for the CH-NAT, CH-PAT, MCI, and AD groups (the Stroop Interference test was not administered to many of the AD participants).

Ten of 36 (28%) CH-NAT participants and 14 of 34 (41%) CH-PAT participants had one or more missing cognitive domain composite z scores and were thus excluded from further analyses. Univariable analyses of cognitive domain composite z-scores are shown in Table 4. For most domain composite scores, there was no evidence of difference between CH-NAT and CH-PAT participants; exceptions were Core Executive (p = 0.004) and Comprehensive Executive (p = 0.01) domains for which CH-NATs performed better than CH-PATs. For multivariable analysis, Language, Encoding, and Delayed Memory domains were not considered as these were highly correlated with other domains that were univariably more related to clinical group. Results of multivariable analysis indicated that only the Core Executive domain was significantly and independently predictive of CH-PAT (OR = 0.15 with CH-NAT as the reference, 95% CI = 0.04–0.65). Univariable analysis of tests that comprise the Core Executive Domain are shown in Table 5; of these, multivariable analysis revealed that only Stroop Interference was significantly and independently predictive of CH-PAT (OR = 0.13 with CH-NAT as the reference, 95% CI = 0.04–0.42, ROC curve in Figure 2). Mean (SD) Stroop Interference z scores for the entire study population (CH, MCI, and AD groups) are shown in Fig. 3.

**Table 4:** Cognitive domains (composite z scores) in the cognitively healthy study population^1^.

Parameter		CH-NAT	CH-PAT	p-value^2^
**Attention**	N	26	20	0.099
	Mean (SD)	0.98 (0.676)	0.58 (0.789)	
	Median	0.83	0.56	
	Min, Max	−0.22, 2.33	−1.00, 1.77	
**Processing Speed**	N	26	20	0.385
	Mean (SD)	0.61 (0.481)	0.50 (0.361)	
	Median	0.59	0.47	
	Min, Max	−0.54, 1.44	0.02, 1.38	
**Language**	N	26	20	0.131
	Mean (SD)	0.98 (0.698)	0.69 (0.519)	
	Median	0.92	0.63	
	Min, Max	−0.29, 2.53	−0.02, 1.86	
**Visuospatial**	N	26	20	0.255
	Mean (SD)	1.07 (0.637)	0.80 (0.735)	
	Median	1.00	0.78	
	Min, Max	0.21, 2.33	−0.44, 2.22	
**Core Executive**	N	26	20	**0.004**
	Mean (SD)	0.71 (0.493)	0.26 (0.519)	
	Median	0.83	0.34	
	Min, Max	−0.74, 1.42	−0.80, 1.24	
**Comprehensive** **Executive**	N	26	20	**0.011**
	Mean (SD)	1.00 (0.483)	0.63 (0.441)	
	Median	0.98	0.57	
	Min, Max	−0.07, 1.78	−0.12, 1.60	
**Encoding**	N	26	20	0.264
	Mean (SD)	0.49 (0.532)	0.28 (0.765)	
	Median	0.58	0.15	
	Min, Max	−0.82, 1.38	−0.70, 1.79	
**Delayed Memory**	N	26	20	0.543
	Mean (SD)	0.74 (0.615)	0.85 (0.555)	
	Median	0.64	0.82	
	Min, Max	−0.43, 2.24	−0.33, 2.03	
**Nonverbal Memory**	N	26	20	0.151
	Mean (SD)	0.44 (0.586)	0.72 (0.708)	
	Median	0.47	0.62	
	Min, Max	−0.97, 1.42	−0.34, 2.07	
**Verbal Memory**	N	26	20	0.488
	Mean (SD)	0.85 (0.754)	0.70 (0.642)	
	Median	0.75	0.73	
	Min, Max	−0.33, 2.58	−0.75, 2.00	

1 Analysis includes only subjects with non-missing data for all domains. Motor domain was excluded due to an excessive number of subjects with missing data.

2 T-test or Wilcoxon rank sum test. CH-NAT = cognitively healthy with normal CSF amyloid/Tau ratio. CH-PAT = cognitively healthy with pathological CSF amyloid/Tau ratio.

**Table 5:** Neuropsychological tests within the core executive domain in the cognitively healthy study population^1^.

Parameter		CH-NAT	CH-PAT	p-value^2^
**D-KEFS** **Tower**	N	26	20	0.115
	Mean (SD)	0.92 (0.796)	0.565 (0.683)	
	Median	0.66	0.66	
	Min, Max	−1.00, 2.33	−0.66, 2.00	
**Trails B**	N	26	20	0.154
	Mean (SD)	0.62 (0.696)	0.212 (1.025)	
	Median	0.87	0.48	
	Min, Max	−1.05, 1.92	−3.02, 1.28	
**Stroop** **Interference**	N	26	20	**0.001**
	Mean (SD)	0.60 (0.516)	−0.001 (0.672)	
	Median	0.62	0.13	
	Min, Max	−0.48, 1.47	−1.28, 1.06	

1 Analysis includes only subjects with non-missing data for all domains. Motor domain was excluded due to an excessive number of subjects with missing data.

2 T-test or Wilcoxon rank sum test. CH-NAT = cognitively healthy with normal CSF amyloid/Tau ratio. CH-PAT = cognitively healthy with pathological CSF amyloid/Tau ratio.

Although all CH participants were asymptomatic with CDRs of 0, we examined the major AD risk factors of blood pressure, fasting lipids and blood sugar (Table 6), as well as their oft-associated MR biomarkers of SVD (Tables 7 and 8), to investigate the possibility that cryptic cerebrovascular disease might confound our analysis, We found no significant differences between the NAT and PAT subgroups. We also examined differences by subgroup in the AD biomarkers of hippocampal volume and N-acetyl aspartate (NAA) as well as myo-inositol (mI) (Table 8). The NAA/mI ratios but not hippocampal volumes were lower in CH-PATs compared to CH-NATs, consistent with underlying AD pathology.

**Table 6:** Cerebrovascular risk factors.

Parameter		CH-NAT	CH-PAT	p-value^1^
**Systolic blood** **pressure**	N	26	20	0.249
	Mean (SD)	135 (18.7)	143 (21.8)	
	Median	134	140	
	Min, Max	93.5, 186	106, 182	
**Diastolic blood** **pressure**	N	26	20	0.176
	Mean (SD)	74.3 (9.66)	78 (9.12)	
	Median	73	80.3	
	Min, Max	52, 92.5	54, 92	
**Cholesterol**	N	25	20	1.000
	Mean (SD)	187 (29.2)	184 (34.1)	
	Median	185	186	
	Min, Max	127, 252	114, 249	
**HDL**	N	25	20	0.631
	Mean (SD)	63.4 (13.6)	64.1 (20.6)	
	Median	63	59	
	Min, Max	36, 83	38, 107	
**LDL**	N	25	20	0.379
	Mean (SD)	112, (28.3)	104 (25.3)	
	Median	106	102	
	Min, Max	57, 179	53, 159	
**VLDL**	N	25	20	0.417
	Mean (SD)	19.8 (9.61)	21.9 (9.87)	
	Median	19	20.5	
	Min, Max	6, 40	11, 44	
**Triglycerides**	N	25	20	0.273
	Mean (SD)	101 (48.1)	114 (47.7)	
	Median	96	107	
	Min, Max	32, 202	55, 220	
**Sugar**	N	25	20	0.855
	Mean (SD)	98 (21.8)	98.7 (21.8)	
	Median	95	93.5	
	Min, Max	77, 176	71, 171	

1 T-test or Wilcoxon rank sum test. CH-NAT = cognitively healthy with normal CSF amyloid/Tau ratio. CH-PAT = cognitively healthy with pathological CSF amyloid/Tau ratio.

**Table 7:** MR imaging.

Small Vessel Disease (SVD)^2^	CH-NAT		CH-PAT		p-value^1^
	N = 22		N = 16		
	Frequency	%	Frequency	%	
**No evidence of SVD**	3	9.7	6	28.6	0.285
**Recent small subcortical infarcts**	3	9.7	1	4.8	
**Lacunes, presumed vascular**	0	0	0	0	
**White matter intensities, presumed vascular**	15	48.4	10	47.6	
**Perivascular spaces**	0	0	0	0	
**Brain atrophy**	10	32.2	4	19.0	

1 Chi-square test.

2 Small Vessel Disease score based on present (0) or absent (1) for each of 5 recommended definitions [28]. CH-NAT = cognitively healthy with normal CSF amyloid/Tau ratio. CH-PAT = cognitively healthy with pathological CSF amyloid/Tau ratio.

**Table 8:** Quantitative volumetric MR imaging and spectroscopy.

		CH-NAT	CH-PAT	p-value^1^
**Hippocampus/Total Brain Volume**	N	22	16	
	Mean (SD)	0.51 (0.098)	0.52 (0.097)	0.303
	Median	0.49	0.53	
	Min, Max	0.38, 0.72	0.37, 0.69	
**NAA/mI ratio**	N	23	16	0.037
	Mean (SD)	2.45 (0.306)	2.24 (0.399)	
	Median	2.39	2.32	
	Min, Max	1.8, 3.31	1.34, 2.92	

1 T-test or Wilcoxon rank sum test. CH-NAT = cognitively healthy with normal CSF amyloid/Tau ratio. CH-PAT = cognitively healthy with pathological CSF amyloid/Tau ratio. NAA/mI = N-acetyl aspartate/myo-inositol.

## Discussion

AD pathology progresses for many years before clinical symptoms are recognized, termed the preclinical phase [4], [5], [57]. We studied this apparently silent preclinical phase in participants with no symptoms in order to assess whether cognitive changes, insufficient to be classified as abnormal, might detect and illuminate this early pathophysiology. Rigorous clinical selection was important in the absence of a diagnostic test in this exploratory and non-longitudinal study. Study participants in our CH-NAT and CH-PAT groups were asymptomatic, had normal neurocognitive testing, normal brain MRI, normal dementia blood workup, and were diagnosed based on CSF Aß_42_ and Tau measures that were within the published ranges [2], [3]. CH-NAT and CH-PAT participants were also similar on commonly recognized confounding variables: age, gender, education, Apoε genotype, as well as use of specific medications and supplements that are typical in older populations. The majority of CH-PAT participants were classified as preclinical AD stage 1 [4]. To explore the best CSF biomarker for AD in both the dementia and preclinical stages, many cases of AD would not have been identified based only on the age-appropriate Aß_42_ levels. In the CH group, 49% had preclinical AD based on the CSF Aß_42_/Tau ratio, a proportion that was consistent with the ratio of preclinical AD in an age-equivalent amyloid imaging study [58]. Sixteen (23%) of the CH group would be classified as preclinical AD if classification had been based only on Aß_42_ levels. The CSF Aß_42_/Tau ratio thus appears to be superior to the CSF Aß_42_ levels individually to identify the dementia and preclinical stages of AD.

Given the large number of neuropsychological tests we administered, we used a two-stage multivariable analysis to identify independently predictive measures of the preclinical condition. We first analyzed cognitive domains, then analyzed the individual tests within those domains that were multivariably significant predictors of preclinical group. Using this approach, the only significant independent predictor of CH-PAT status was the color-word Stroop Interference. Our result was unexpected, as most studies of early AD report a memory dysfunction rather than an executive dysfunction [59]–[61]. Nevertheless, while memory disturbance is the most noticeable feature in established AD, a rising number of studies report non-amnestic changes preceding memory disturbance. For instance, non-amnestic MCI is a category of MCI, from which some progress to AD [62]. A longitudinal study of the transition from healthy aging to AD revealed changes in visuospatial testing before memory, prior to dementia(Johnson et al., 2009); brain atrophy rates were correlated with low CSF Aß_42_ and impaired executive function (Trails B) in the absence of a memory dysfunction in a study of older CH adults [63]. Another study found that specific measures of executive function, including inhibition, predicted cognitive decline in both normal controls and those with MCI or dementia [25]. Our results are even more consistent with a study that reported an increased Stroop coefficient of variability with age and in early AD compared to non-early AD patients; this variability also correlated with CSF Aß_42_ and Tau levels and was accentuated in healthy Apoε-4 carriers [64]. Furthermore, errors in color naming in the Stroop, but not deterioration in declarative memory, have been shown to be good predictors of conversion to dementia [65] and a combination of the Stroop task with task switching was reported to be the best discriminator from a psychometric battery of healthy aging versus AD [66]. Our preclinical group differed from study participants in these reports in that participants from our study generally had lower Apoε genotype scores. Moreover, our participants had no memory impairment, were diagnosed on their CSF Aß_42_/Tau ratio, and were all asymptomatic. Overall, the decrease in executive performance without loss of memory that we observed in participants classified as preclinical AD adds to the expanding evidence that measurable decrease in executive function precedes deterioration of memory in preclinical AD. Most notable is the predictive potential of the Stroop Interference test.

To assess the underlying pathways involved in the response inhibition as captured on the Stroop Interference test, a meta-analysis of neuroimaging data between 1990 and 2005 of interference tasks including Stroop found that involvement of the bilateral dorsolateral prefrontal cortex, inferior frontal gyrus, anterior cingulate cortex, and posterior parietal cortex were most consistently reported [67]. Therefore, poorer performance by the CH-PAT subgroup on the Stroop Interference suggests that there is prefrontal cortical dysfunction in preclinical AD.

Subcortical ischemic vascular disease is associated with executive dysfunction [68] and white matter hyperintensities are associated with amyloid deposits as visualized by positron emission tomography [69]. Although our population was asymptomatic with CDRs of zero, we investigated ischemic risk factors and MRI evidence of SVD to investigate whether subtle cerebrovascular disease status may have differed between CH subgroups, thus confounding our analysis of Stroop Interference. None of these factors were related strongly enough to CH subgroup to create a confounding effect. However, we observed a lower NAA/mI ratio, an accepted biomarker of AD [70], in CH-PATs compared to CH-NATs; this, along with the lower CSF Aß_42_/Tau ratio used to classify the CH-PAT subgroup, provide support that preclinical AD pathology rather than cerebrovascular disease is most likely responsible for the altered executive function.

We found that executive function but not memory deteriorates in our preclinical subgroup. Since impaired memory is inevitable in AD, we fully expect that our CH-PAT subgroup will develop memory disturbances. However, the few studies of preclinical AD have mixed results on executive and memory changes, e.g., [25], [26], most likely due to heterogeneity within and between the different populations in each study. Conflicting findings are perhaps predictable since there is no consensus test for preclinical AD. Moreover, memory and executive functions are closely linked, as emphasized by a report of impairment in dual task performance in early AD [71]. These authors also show that memory capacity is spared via minimal interference in those with amnestic MCI that progress to AD [72]. We conclude that the neuropsychological functions of cognitively healthy older people require substantially further definition for a better understanding of normal aging and preclinical AD.

Our finding that the Stroop Interference improves the prediction of preclinical disease defined by the CSF Aß_42_/Tau ratio contributes to important issues regarding preclinical AD. First, these findings may improve our understanding of the pathology. The hippocampus is the primary origin of the intraneuronal neurofibrillary pathology in AD, and it is the most severely affected brain location based on neuronal counts [73], [74]. Furthermore, failing memory, the commonly recognized presentation of AD, is largely a result of hippocampal dysfunction. Extracellular amyloid deposits, on the other hand, start in the basilar isocortex, are seen before any intraneuronal neurofibrillary changes, and do not initially involve the hippocampus [19]. Positron emission tomography with two different approaches that identify amyloid corresponds with this pathological distribution *in vivo* and identifies increased amyloid in preclinical AD [22], [75]. Therefore, since we find that the first functional change is a decline in executive but not memory performance, this is most consistent with dysfunction from extracellular amyloid deposits in the basilar isocortex preceding the intraneuronal neurofibrillary pathology and hippocampal dysfunction [19]. A functional imaging study [76] in young (mean 33 years) and older (mean 62 years) cognitively healthy adults is consistent with this pathology [19]; these authors reported increased activation in the dorso-lateral prefrontal cortex with decreased deactivation in the posterior cingulate during working memory and incidental episodic encoding memory testing, features that coincided with poorer performance in aging. Until Tau imaging can be investigated in vivo [23], [24], we conclude that the temporal progression of the CSF Aß_42_ biomarker and the spatial and temporal evolution of extracellular amyloid pathology are more directly correlated with our preclinical AD data than that of the later temporal change of the CSF Tau biomarker and the spatial and temporal evolution of the intraneuronal neurofibrillary pathology.

The second important issue in preclinical AD related to our result is that further diagnostic criteria for preclinical AD are still needed. Though our results are more directed towards research than clinical practice, clinicians may consider adding the simple-to-administer Stroop Interference test with the recently validated AD8 [77] to screen for preclinical AD and to monitor therapy.

Third, awareness of the potential for subtle impaired executive function in asymptomatic older adults may be helpful for individuals and/or families in recognizing and managing the early stages of this progressive disorder. For example, catastrophic financial decisions are a well-recognized consequence of AD that precedes diagnosis [78], and earlier diagnosis may help the family avoid such events.

Finally, physical exercise has been reported to increase prefrontal oxygenation while also improving performance on the Stroop Interference [79]. This raises the possibility that factors that improve Stroop performance, such as exercise, may also impede progression to dementia. It is interesting that our preclinical AD participants, the CH-PATs, reported less physical activity than the CH-NATs; however, CH-PATs also had somewhat higher BMI than CH-NATs, which could also explain an association between low physical activity and preclinical AD in our data. The relationships between preclinical AD, physical activity, BMI, and the Stroop Interference performance merit further study to disentangle the possible effects of exercise and BMI on Stroop Interference performance and ultimately on the risk of preclinical AD, particularly as defined by CSF biomarkers.

There were limitations to our study. Our minimum age among CH participants was 68 years and a younger population will be required to investigate earlier pathophysiology. Pathophysiological progression was not characterized by longitudinal study and we had no pathological verification of disease status (except in one case). We used the same study population to both predict group membership (based on CSF Aß_42_/Tau) and to analyze group differences; i.e., we did not attempt to internally validate the cutoff, likely resulting in CH-NAT/CH-PAT classification error. Some participants were excluded from multivariable analysis due to missing neuropsychological test results. Reasons for missing test results were data being inadvertently omitted, score sheets not being available, or participants ending the testing prematurely for personal reasons. Compared to participants with complete test results, these participants were more likely to be CH-PAT rather than CH-NAT, male, and not on certain medications. Thus, it is possible that gender and/or other factors related to medication use affects neuropsychological test performance in such a way that excluding these participants biased our results. Our study was exploratory and, as such, control of type 1 error, while considered, was not rigorously implemented. These limitations are important to take into account, not only when interpreting our conclusions but also when designing future studies to confirm and further explore our findings.
